# Differential activation of endocrine-immune networks by arthritis challenge: Insights from colony-specific responses

**DOI:** 10.1038/s41598-017-00652-4

**Published:** 2017-04-06

**Authors:** Tamara S. Bodnar, Matthew D. Taves, Katie M. Lavigne, Todd S. Woodward, Kiran K. Soma, Joanne Weinberg

**Affiliations:** 1 Department of Cellular and Physiological Sciences, University of British Columbia, Vancouver, BC Canada; 2 Department of Psychology, University of British Columbia, Vancouver, BC Canada; 3 Department of Zoology, University of British Columbia, Vancouver, BC Canada; 4 Department of Psychiatry, University of British Columbia, Vancouver, BC Canada; 5 Translational Research Unit, BC Mental Health and Addictions Research Institute, Provincial Health Services Authority, Vancouver, BC Canada; 6 Graduate Program in Neuroscience, University of British Columbia, Vancouver, BC Canada; 7 Djavad Mowafaghian Centre for Brain Health, University of British Columbia, Vancouver, BC Canada

## Abstract

Rheumatoid arthritis (RA) is a chronic inflammatory condition with variable clinical presentation and disease progression. Importantly, animal models of RA are widely used to examine disease pathophysiology/treatments. Here, we exploited known vendor colony-based differences in endocrine/immune responses to gain insight into inflammatory modulators in arthritis, utilizing the adjuvant-induced arthritis (AA) model. Our previous study found that Sprague-Dawley (SD) rats from Harlan develop more severe AA, have lower corticosteroid binding globulin, and have different patterns of cytokine activation in the hind paw, compared to SD rats from Charles River. Here, we extend these findings, demonstrating that Harlan rats show reduced hypothalamic cytokine responses to AA, compared to Charles River rats, and identify colony-based differences in cytokine profiles in hippocampus and spleen. To go beyond individual measures, probing for networks of variables underlying differential responses, we combined datasets from this and the previous study and performed constrained principal component analysis (CPCA). CPCA revealed that with AA, Charles River rats show activation of chemokine and central cytokine networks, whereas Harlan rats activate peripheral immune/hypothalamic-pituitary-adrenal networks. These data suggest differential underlying disease mechanism(s), highlighting the power of evaluating multiple disease biomarkers, with potential implications for understanding differential disease profiles in individuals with RA.

## Introduction

Rheumatoid arthritis (RA) is a chronic inflammatory condition with an etiology that is not yet well understood. While considered an autoimmune disease, there is no clear consensus as to the exact triggers of RA, and a growing list of possible autoantigens and infectious agents, as well as environmental conditions has been associated with RA^1–3^. There is also variability in prevalence and incidence^4^ as well as the clinical presentation of RA^5^. Even in patients with significant overlap in RA symptoms, dissimilar physiological profiles, with differential cytokine expression patterns, leukocyte infiltration and involvement, and synovial damage is common^6, 7^. Given this inherent variability, it is not surprising that there is also a variable response to treatment^8^.

It is known that RA is exacerbated by chronic stress^9^, although the mechanisms by which this occurs remain unclear. Although glucocorticoids are known to be involved in RA, both immunosuppressive and immunostimulating effects of glucocorticoids have been reported^10^. Alterations in hypothalamic-pituitary-adrenal (HPA) reactivity have, however, been shown to be a critical factor in experimentally-induced arthritis^11, 12^, as well as in a subset of cases of RA. Relative adrenal insufficiency, inappropriate activation of the HPA axis resulting in an impaired ability to inhibit ongoing inflammation^13^, and other forms of HPA axis dysregulation have been observed to varying degrees in patients with RA^14^. Yet a clear HPA deficit has yet to be identified^15^, and it has been suggested that investigations into the HPA response, in concert with immune markers (cytokines), would be more informative than continued probing of the HPA axis alone^16, 17^.

Peripheral cytokines are integral in RA pathology^18^ and there is also evidence of increased cytokine levels in the brain^19^. Importantly, RA and other conditions associated with chronic inflammation are known risk factors for mental illness^20^, likely mediated, at least in part, by cytokine disturbances. While the mechanisms are not fully understood, there is evidence that the blood-brain barrier is compromised in RA^21^ and in animal models of experimentally-induced arthritis^22^. TNF-α, a key cytokine in RA pathogenesis, likely plays a role in neuroinflammation with RA^19^, as some of the neuropsychiatric features associated with the disorder, such as fatigue and central sensitization to pain, may be dampened with anti-TNF agents^23, 24^. However, further investigation into the role of the numerous other cytokines involved in RA-mediated neuroinflammation, and how cytokine levels vary by disease state, is needed.

We have exploited known vendor colony-based differences in endocrine/immune responses to elucidate endocrine and immune mechanisms underlying RA, utilizing the adjuvant-induced arthritis (AA) model, a well-established model of human RA. The response to AA was investigated in female subjects due to the increased rates of autoimmune disorders in women compared to men^25^, and previously reported heightened sensitivity to experimentally induced arthritis in female rodents^26^. Sprague Dawley (SD) rats from two different vendors – Harlan and Charles River – were utilized as a tool to probe the possible basis for the variability in RA course. We showed previously^27^ that colony of origin impacts the AA disease course. Compared to Charles River rats, we found that Harlan rats have an increased incidence and severity of AA, lower corticosteroid binding globulin (CBG) despite similar circulating corticosterone levels, and different patterns of cytokine activation in the hind paw.

The current study builds on these findings to extend our investigation of endocrine (corticosterone) and immune (cytokines) parameters in key immune compartments, including the paws, the main site of inflammation in the AA model, spleen, thymus, popliteal lymph nodes, and brain in these same animals. Following investigation of these individual tissue compartments (univariate analyses), and to probe for networks of endocrine/immune variables underlying these differential responses, we combined all of our measures from the current and previous study, which resulted in a dataset with a total of 44 variables suitable for network analysis, and performed a constrained principal component analysis (CPCA). CPCA is a multivariate technique combining multiple regression and principal component analysis (PCA) into a unified framework^28^, which has the advantage of being able to relate the networks back to the independent variables. While CPCA has been used in a wide variety of fields^29–32^ to the best of our knowledge, this is the first time CPCA has been used to examine endocrine and immune parameters. This technique is particularly well suited to the examination of endocrine and cytokine activity and interactions, as the complex balance of variables, rather than changes in a single variable, is most relevant to disease pathophysiology. Importantly, our multi-systems approach is critical for better understanding of the underlying pathophysiology in complex diseases such as RA^33^. We propose that utilizing colony-based differences together with a network approach in assessing endocrine and immune responses to an inflammatory challenge not only provide novel information on key modulators or mediators of inflammation, but also a more complete and nuanced clinically-relevant representation of factors influencing disease incidence and course, that will have potential implications for understanding clinical RA and other autoimmune disorders.

## Results

### Analysis of estrous cycle

Due to the potential modulatory role of estrous cycle on levels of endocrine and immune markers, estrous cycle was staged at the end of the experimental period (day 16 post-injection, the termination day). Differences in the proportion of rats within each stage of the estrous cycle (proestrus, estrous, diestrus) by either colony or arthritis severity state were not detected, with >90% of rats in diestrus at the time of termination. Thus, data were not further stratified by estrous stage for the subsequent analyses.

### Local corticosterone levels increased in the joints and immune tissues with severe arthritis

**Figure 1:**
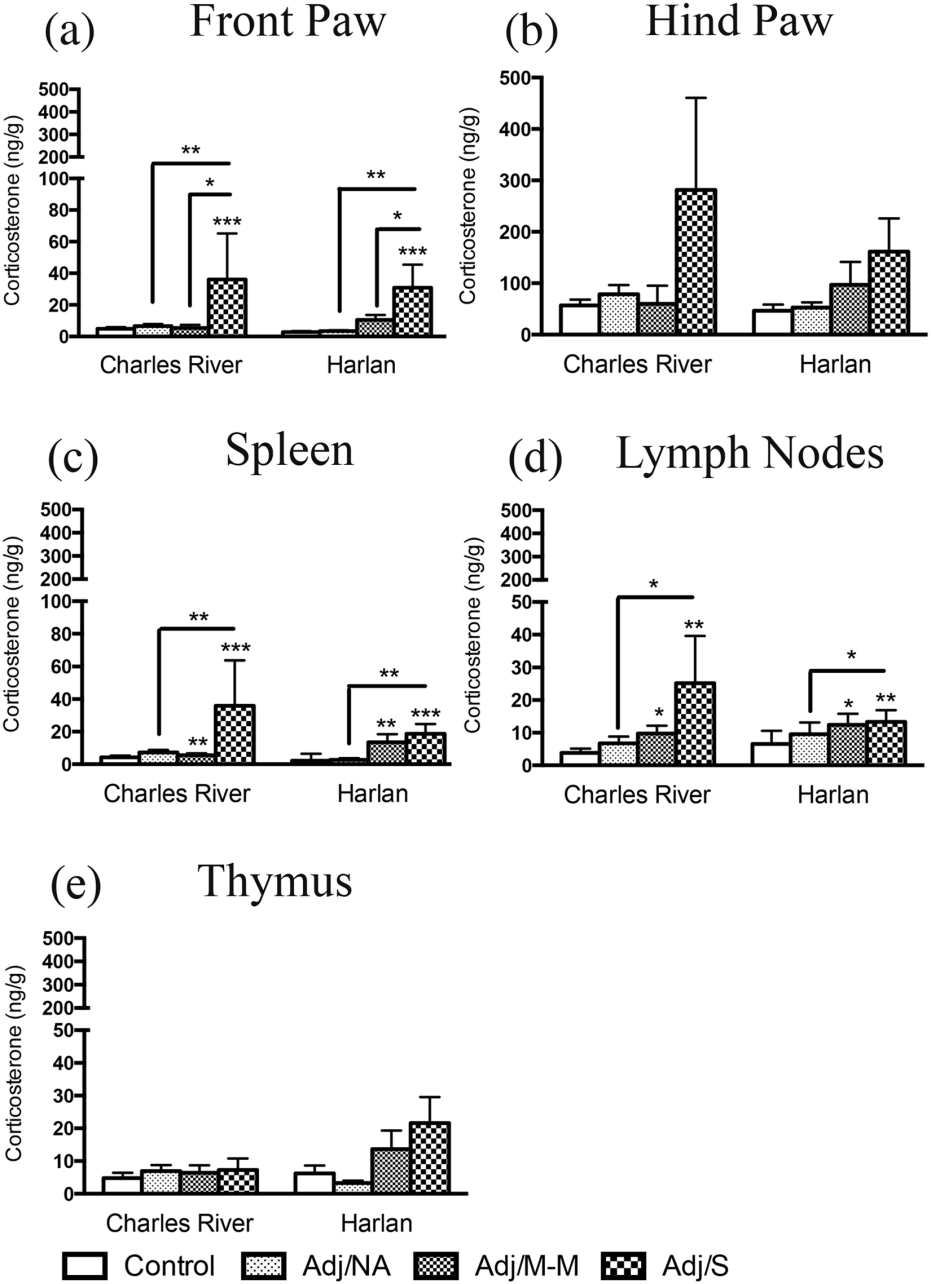
Local corticosterone levels in the paws and immune tissues. Bars represent mean corticosterone level ± SEM. Data are presented as ng corticosterone/g tissue with statistical analysis conducted on Blom transformed (normalized) data. The presence of an asterisk (*) indicates a main effect of AA severity, with the comparison to the control group, unless otherwise indicated. *Post hoc:* *p < 0.05; **p < 0.01; ***p < 0.001. Control: saline-injected; Adj/NA: no clinical signs of AA following CFA injection; Adj/M-M: mild-moderate AA, clinical score ≥1, <8; Adj/S: severe AA, clinical score ≥8.

Corticosterone, which has anti-inflammatory properties, was measured in joints (front and hind paw, the tissue most affected in the AA model) and immune tissues (thymus, spleen, popliteal lymph nodes) to assess whether differences in corticosterone levels could help explain colony differences in the response to AA challenge (Fig. 1). Corticosterone levels in the front paw differed by AA severity [main effect of AA severity: F_(3,49)_ = 5.38, *p* = 0.003], with levels increasing in both Charles River and Harlan rats with severe AA (Adj/S), as expected (Fig. 1a). Overall, the pattern of corticosterone in the hind paw was similar to that in the front paw, although the ANOVA failed to reach significance [AA severity: F_(3,48)_ = 2.28, *p* = 0.091] (Fig. 1b). Of note, however, inspection of Fig. 1b reveals that the overall trend for increased corticosterone is driven by the Adj/S group of Charles River rats. In the spleen and popliteal lymph nodes, corticosterone levels increased with development of AA (Adj/M-M, Adj/S) in rats from both colonies [main effects of AA severity – spleen: F_(3,48)_ = 6.95, *p* = 0.001; popliteal lymph nodes: F_(3,43)_ = 4.60, *p* = 0.007; Fig. 1c,d). Corticosterone levels did not differ by AA severity or colony in the thymus (Fig. 1e).

### Splenic levels of proinflammatory cytokines increased with AA in both Charles River and Harlan rats

**Figure 2:**
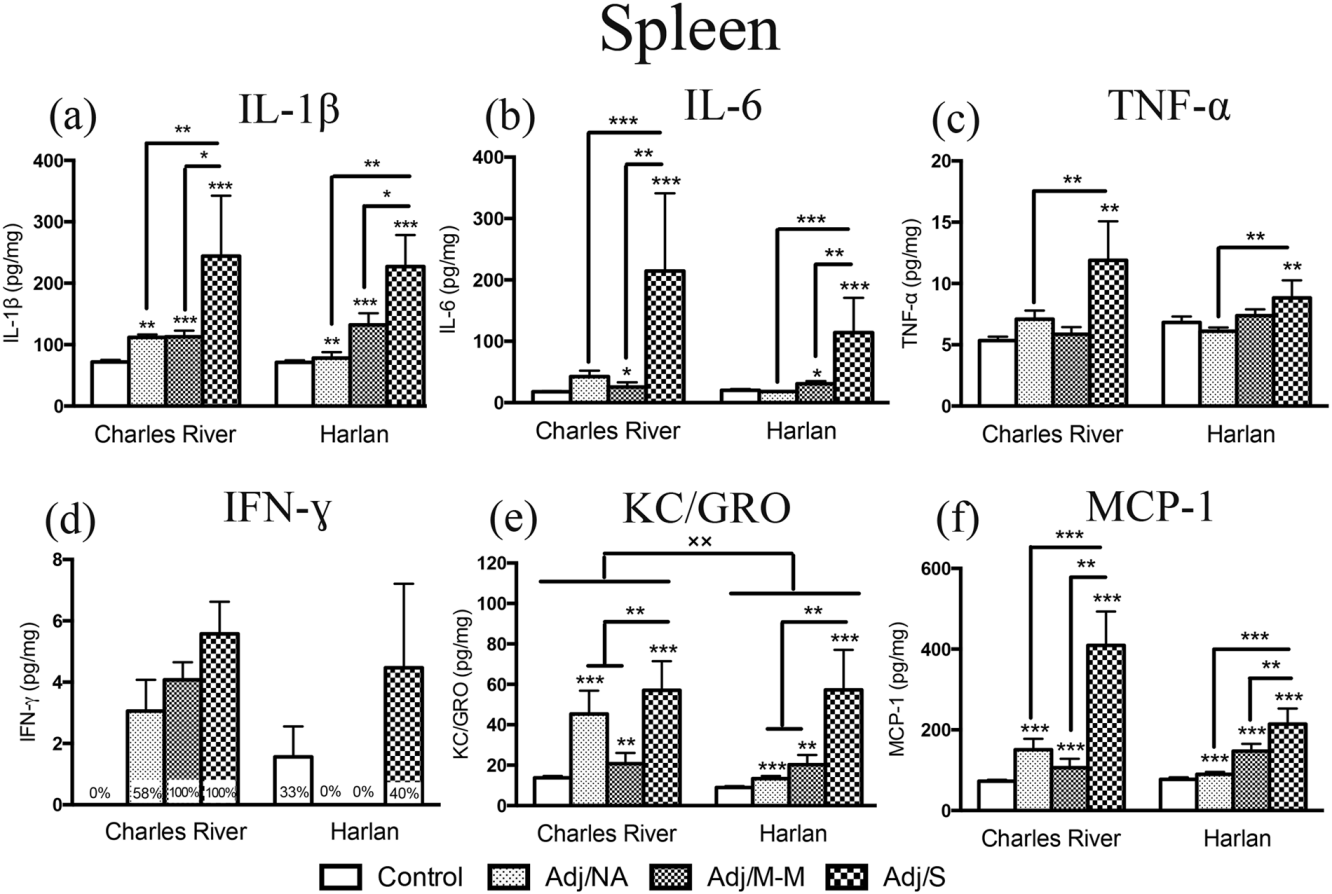
Cytokine levels in the spleen. Bars represent mean cytokine level ± SEM. Data are presented as pg cytokine/mg protein with statistical analysis conducted on Blom transformed (normalized) data. The presence of an asterisk (*) indicates a main effect of AA severity, with the comparison to the control group, unless otherwise indicated. The “×” symbol indicates a significant main effect of colony. Percentages on bars indicate the percent of rats with detectable cytokine levels, (within each AA severity state) and these data (d) were not analyzed statically due to non-normal distribution. *Post hoc:* *p < 0.05; **^/××^ *p* < 0.01; ***p < 0.001. Control: saline-injected; Adj/NA: no clinical signs of AA following CFA injection; Adj/M-M: mild-moderate AA, clinical score ≥1, <8; Adj/S: severe AA, clinical score ≥8.

Cytokine levels were assessed in the spleen, a critical immune organ and important inflammatory site in the AA model. Splenic levels of IL-1β, IL-6, TNF-α, KC/GRO, and MCP-1 increased with AA in rats from both Charles River and Harlan colonies [main effects of AA severity: IL-1β: F_(3,46)_ = 14.53, *p* < 0.001; IL-6: F_(3,48)_ = 9.90, *p* < 0.001; TNF-α: F_(3,49)_ = 3.88, *p* = 0.014; KC/GRO: F_(3,48)_ = 12.69, *p* < 0.001; MCP-1: F_(3,49)_ = 17.28, *p* < 0.001;]. IL-1β, KC/GRO, and MCP-1 levels increased in all CFA-injected rats, whether or not they showed clinical signs of inflammation, with the highest levels in rats that developed severe AA (Adj/S) (Fig. 2a,e,f). Comparatively, levels of IL-6 and TNF-α only increased above control levels with the development of mild-moderate and/or severe AA (Fig. 2b,c). Furthermore, levels of KC/GRO were higher overall in Charles River compared to Harlan rats [main effect of colony – F_(1,48)_ = 10.40, *p* = 0.002]. Levels of IFN-ɣ were undetectable in at least one severity state and thus were not analyzed statistically (Fig. 2d; percent of detectable samples indicated). Of note, 100% of Charles River rats that developed arthritis (Adj/M-M, Adj/S) had detectable levels of IFN-ɣ in the spleen, compared to 0% of Adj/M-M and 40% of Adj/S for Harlan rats. Levels of the anti-inflammatory cytokines IL-4 and IL-10 were low or below the limit of detection for the majority (>50%) of rats from both colonies.

### Hypothalamic cytokine levels increased to a greater extent in Charles River compared to Harlan rats with AA

Levels of key pro- and anti-inflammatory cytokines were measured in the hypothalamus, an important integrator of both endocrine and immune responses. Cytokines stimulate the HPA axis following an immune challenge, with downstream effects on hormone release at both the hypothalamus and pituitary gland^34^, ultimately impacting immune function and resolution from inflammation. It was hypothesized that colony differences in the hypothalamic cytokine response may be a key factor underlying the differential response of Charles River and Harlan rats to AA.

**Figure. 3:**
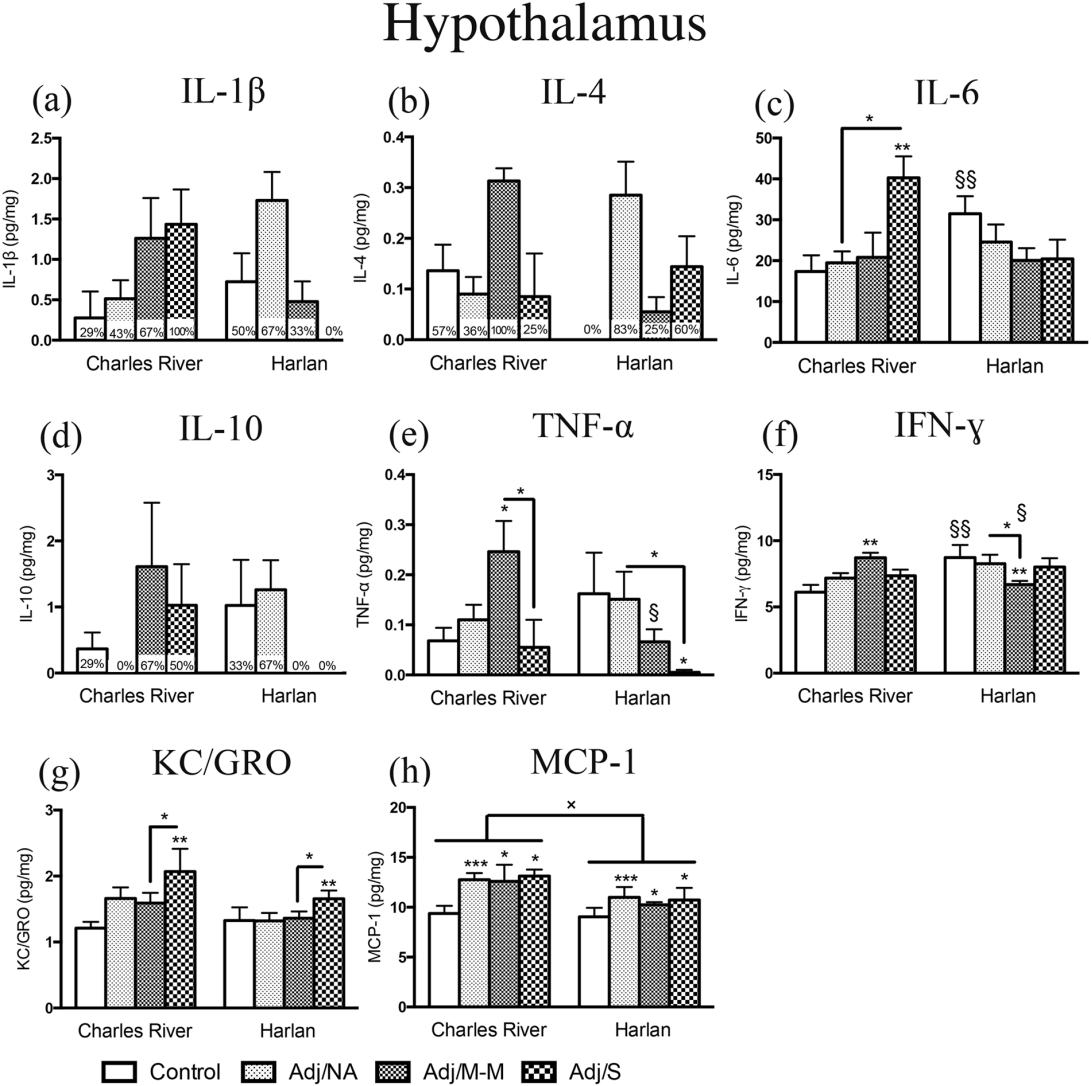
Cytokine levels in the hypothalamus. Bars represent mean cytokine level ± SEM. Data are presented as pg cytokine/mg protein with statistical analysis conducted on Blom transformed (normalized) data. The presence of an asterisk (*) indicates a main effect of AA severity, with the comparison to the control group, unless otherwise indicated. The “×” symbol indicates a significant main effect of colony. The “§” symbol indicates a significant colony × AA severity interaction, with the symbol denoting a comparison between colonies, within rats of the same AA severity state. Percentages on bars indicate the percent of rats with detectable cytokine levels, (within each AA severity state) and these data (a, b, d) were not analyzed statically due to non-normal distribution. *Post hoc:* *^/§/×^ *p* < 0.05; **^/§§^ *p* < 0.01; ***p < 0.001. Control: saline-injected; Adj/NA: no clinical signs of AA following CFA injection; Adj/M-M: mild-moderate AA, clinical score ≥1, <8; Adj/S: severe AA, clinical score ≥8.

We found colony differences in cytokine levels of both control (saline-injected) and AA animals. In the control condition, Harlan had higher levels of IL-6 and IFN-ɣ than Charles River rats (Fig. 3c,f). By contrast, in response to CFA injection, we found significant interactions between colony and AA severity for TNF-α, IFN-ɣ, and IL-6 [TNF-α: F_(3,48)_ = 2.75, *p* = 0.05; IFN-ɣ: F_(3,49)_ = 4.81, *p* = 0.005; IL-6: F_(3,49)_ = 3.82, *p* = 0.016]. Levels of TNF-α and IFN-ɣ increased with mild-moderate AA (Adj/M-M) in Charles River but not Harlan rats (Fig. 3e,f), and thus were lower in Harlan compared to Charles River in the Adj/M-M condition. Furthermore, IL-6 levels increased with severe AA (Adj/S) in Charles River rats, whereas in Harlan rats, mean hypothalamic IL-6 levels were highest under control conditions (Fig. 3c).

For the chemokines examined, CFA injection increased MCP-1 levels in both Charles River and Harlan rats compared to their control counterparts [main effect of AA severity: F_(1,47)_ = 6.03, *p* = 0.018] (Fig. 2h). As a group, however, Harlan had lower overall MCP-1 levels than Charles River rats [main effect of colony: F_(3,47)_ = 4.32, *p* = 0.009]. Comparatively, KC/GRO increased with severe AA in rats from both colonies [main effect of AA severity: F_(3,49)_ = 3.51, *p* = 0.022] (Fig. 3g).

Levels of IL-1β, IL-4, and IL-10 were undetectable in at least one condition and thus not analyzed statistically (Fig. 3a,b,d; percent of detectable samples indicated). Of note, for Charles River, the highest mean levels of IL-1β, IL-4, and IL-10 were detected in rats that developed AA (Adj/M-M or Adj/S conditions), whereas, for Harlan, the highest mean cytokine levels were in those that failed to develop clinical signs of inflammation (Adj/NA). Surprisingly, no Harlan rats with severe AA (Adj/S) had detectable levels of IL-1β or IL-10.

### Hippocampal cytokine levels were mildly affected by AA, with colony differences in overall levels of IFN-ɣ and KC/GRO

**Figure 4:**
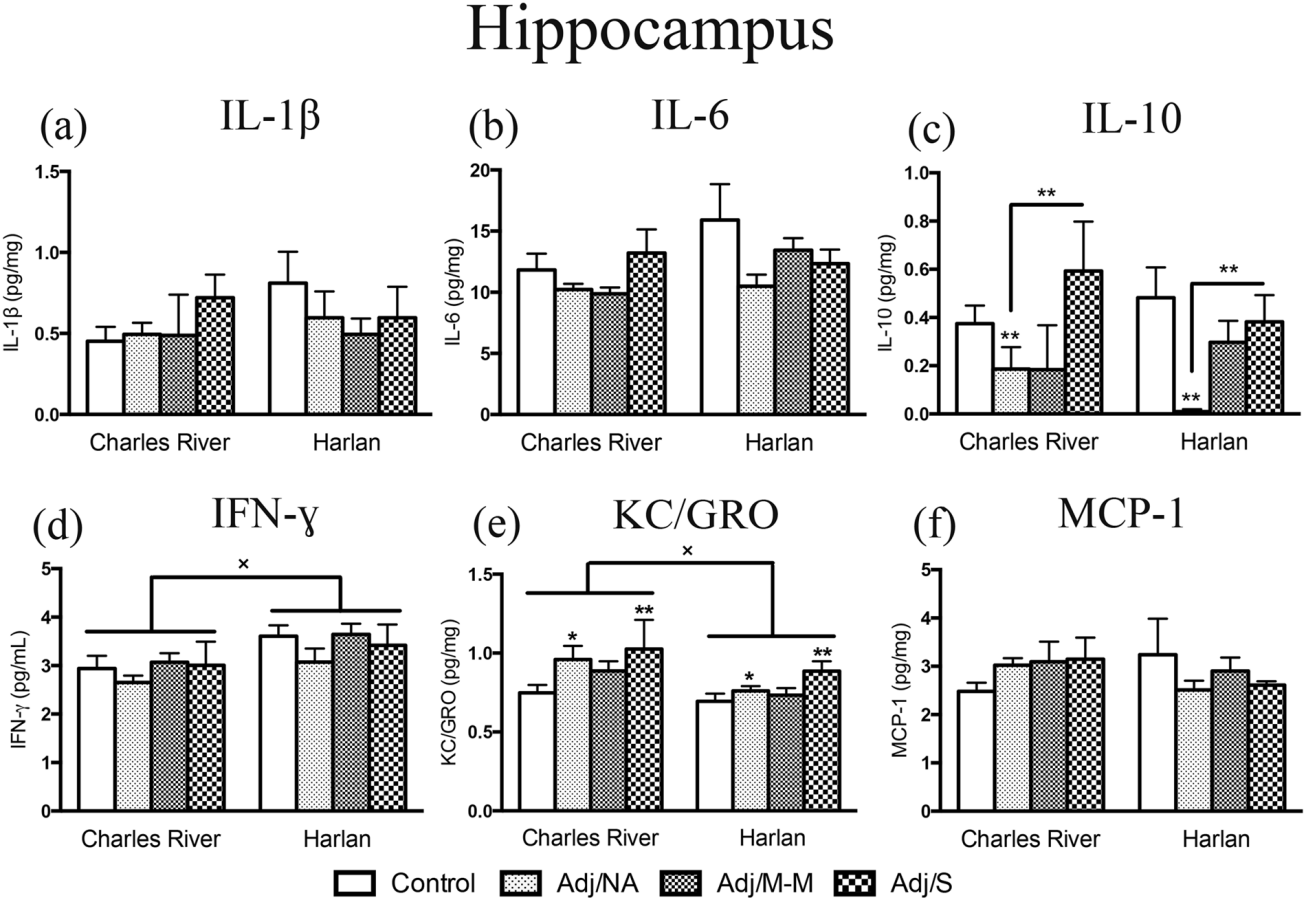
Cytokine levels in the hippocampus. Bars represent mean cytokine level ± SEM. Data are presented as pg cytokine/mg protein with statistical analysis conducted on Blom transformed (normalized) data. The presence of an asterisk (*) indicates a main effect of AA severity, with the comparison to the control group, unless otherwise indicated. The “×” symbol indicates a significant main effect of colony. *Post hoc:* *^/×^ *p* < 0.05; **p < 0.01. Control: saline-injected; Adj/NA: no clinical signs of AA following CFA injection; Adj/M-M: mild-moderate AA, clinical score ≥1, <8; Adj/S: severe AA, clinical score ≥8.

The hippocampus contains one of the highest densities of proinflammatory cytokine receptors in the brain [reviewed in^35^], and cytokine levels were assessed to probe for differential neuroimmune responses to AA between colonies. We found that IFN-ɣ levels were higher, and KC/GRO levels were lower, in Harlan compared to Charles River rats in the control condition [main effects of colony – IFN-ɣ: F_(1,48)_ = 6.58, *p* = 0.013; KC/GRO: F_(1,48)_ = 4.19, *p* = 0.046] (Fig. 4d,e). In addition, only two cytokines responded to CFA injection, independent of colony: KC/GRO levels [F_(3,48)_ = 3.01, *p* = 0.039] were increased in the Adj/NA and Adj/S conditions, while IL-10 levels [F_(3,47)_ = 4.72, *p* = 0.006] were decreased in the Adj/NA compared to the control and Adj/S conditions (Fig. 4e,c).

**Figure 5:**
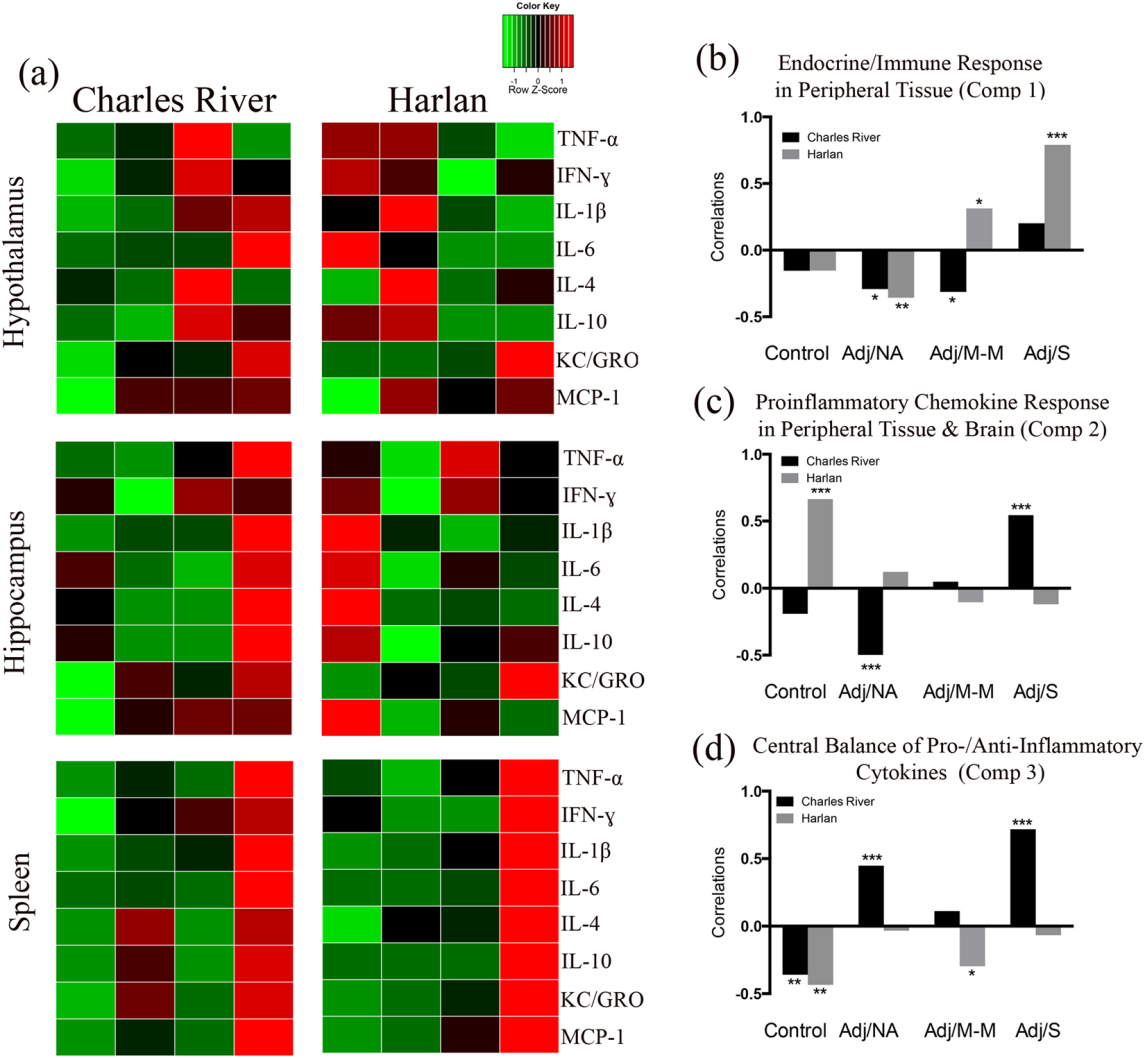
Endocrine/immune activation in response to complete Freund’s adjuvant (CFA) injection. (**a**) Heatmaps showing the overall cytokine response to CFA-injection in the hypothalamus, hippocampus, and spleen. Columns represent severity states (control, Adj/NA, Adj/M-M, Adj/S), split by colony, as indicated. Rows represent mean cytokine levels (z-scored data) by severity state, within each tissue. Colors indicate deviations from the mean of zero, as indicated in the colour key. (**b–d**) Graphical representation of the correlations between the experimental condition (colony, AA severity) and the subjects’ component scores for component 1 (*Endocrine/Immune Response in Peripheral Tissue*), 2 (*Proinflammatory Chemokine Response in Peripheral Tissue & Brain*), and 3 (*Central Balance of Pro-/Anti-Inflammatory Cytokines*) from the CPCA. *p < 0.05; **p < 0.01; ***p < 0.001. Control: saline-injected; Adj/NA: no clinical signs of AA following CFA injection; Adj/M-M: mild-moderate AA, clinical score ≥1, <8; Adj/S: severe AA, clinical score ≥8.

Levels of IL-1β, IL-6, and MCP-1 did not differ by AA status or vendor. Notably, however, similar to what was observed in the hypothalamus, in Charles River rats, mean levels of IL-1β, IL-6, and MCP-1 were highest in rats that developed severe arthritis (Adj/S) whereas in Harlan rats, mean cytokine levels were highest in controls (Fig. 4a,b,f). Hippocampal levels of IL-4 and TNF-α were low or below the limit of detection for the majority (>50%) of rats (data shown in cytokine heatmaps only – Fig. 5).

### The global cytokine profile following CFA injection differed between Charles River and Harlan rats

To highlight the overall cytokine pattern, we integrated the cytokine responses to CFA injection in the hypothalamus, hippocampus, and spleen, and depicted these in a heatmap (Fig. 5). This heatmap was built using z-score data, averaging cytokine levels by AA severity state and colony. As a whole, in the spleen, the profile between colonies was comparable, with severely arthritic rats demonstrating the greatest cytokine increases. By contrast, in the brain, cytokine activation patterns in response to CFA injection differed between colonies. For Charles River rats in general, hypothalamic cytokine levels were highest in rats that showed clinical signs of AA (Adj/M-M, Adj/S) and hippocampal cytokine levels were highest in rats that developed severe AA (Adj/S). An opposing profile was detected in Harlan rats, where cytokine levels were generally highest in controls (in both hypothalamus and hippocampus) and rats that failed to develop clinical signs of AA (Adj/NA) (in hypothalamus). Of note, similar profiles were detected for both pro- and anti-inflammatory cytokines – the two “opposing” groups of cytokines generally increased or decreased together, as can be seen in Fig. 5a.

### CPCA analysis indicates that Charles River and Harlan rats rely on different endocrine/immune networks throughout the course of AA

In order to extend beyond individual analytes and to probe for networks of endocrine and immune parameters that are differentially activated in the two colonies, CPCA, a novel data reduction technique, was utilized. As noted, to assess comprehensive networks, the present data set was combined with our previous data set from these same animals^27^ (previous data include hind paw and plasma cytokines, and plasma corticosterone, ACTH, CBG; previously reported measures are highlighted in blue in Table 1). Briefly, CPCA involves first regressing the matrix of dependent variables (i.e., z-score transformed endocrine and inflammatory measures) on the independent variables (i.e. colony and AA severity), resulting in a matrix of predicted scores reflecting the variance in endocrine and inflammatory measures that is predictable from colony and AA severity, referred to as the predictable variance. In the current analysis, the experimental model (i.e. colony and AA severity) accounted for 28.06% of the total variance in the parameters examined. A principal component analysis (PCA) was then performed on the endocrine/immune variance constrained to that predictable from colony and AA severity, which revealed a three-component solution. The component loadings for all of the parameters, for each of the three components, are listed in Table 1. The first component explained 34.87% of the predictable variance (9.79% of the total variance), the second component explained 26.69% of the predictable variance (9.17% of the total variance), and the third component explained 15.09% of the predictable variance (4.23% of the total variance). Each components was defined based on the endocrine/immune parameters that showed the highest loadings for that component (Table 1): Component 1 was defined as *Endocrine/Immune Response in Peripheral Tissue*, as it primarily included cytokines in the hind paw and corticosterone levels in plasma, paws, lymph nodes, and spleen; Component 2 was defined as *Proinflammatory Chemokine Response in Peripheral Tissue & Brain*, as it included primarily KC/GRO and MCP-1 in plasma, spleen, and brain; and Component 3 was defined as *Central Balance of Pro-/Anti-Inflammatory Cytokines*, as it included both pro-/anti-inflammatory cytokines (IL-6, IL-4, IL-10) exclusively within the brain.

**Table 1:** Component loadings for the endocrine/immune variance constrained to that predictable from colony and AA severity. Networks are interpreted using loadings set in bold font.

Variables	*Comp1*	*Comp2*	*Comp3*
*Hind paw IL-1β*	**0.65**	**0.36**	0.13
*Hind paw MCP-1*	**0.64**	0.33	0.09
*Hind paw TNF-α*	**0.57**	0.31	−0.10
*Hind paw KC/GRO*	**0.53**	**0.43**	0.15
*Hind paw IL-6*	**0.53**	0.18	0.09
*CBG*	**−0.49**	−0.18	**−0.37**
*Plasma TNF-α*	**0.46**	**0.41**	−0.11
*Hind paw IFN-ɣ*	**0.45**	0.05	0.12
Spleen CORT	**0.43**	0.08	0.00
*Plasma CORT*	**0.43**	0.16	−0.14
Front paw CORT	**0.41**	0.27	0.13
Popliteal lymph node CORT	**0.37**	0.07	0.02
Hypothalamus TNF-α	**−0.34**	−0.02	0.11
*Plasma KC/GRO*	0.15	**0.65**	0.17
Spleen MCP-1	**0.40**	**0.59**	0.19
*Plasma IL-6*	**0.39**	**0.55**	0.19
*Plasma IL-1β*	0.03	**0.44**	0.22
Hypothalamus KC/GRO	0.13	**0.43**	0.03
*Plasma MCP-1*	**0.38**	**0.42**	0.18
Spleen KC/GRO	0.29	**0.41**	−0.15
Hypothalamus MCP-1	−0.07	**0.40**	−0.05
Spleen IL-6	0.31	**0.40**	0.13
Hippocampus KC/GRO	0.06	**0.40**	−0.12
Spleen IL-1β	0.31	**0.39**	−0.09
Spleen TNF-α	**0.35**	**0.37**	0.22
Spleen IFN-ɣ	0.03	**0.36**	−0.12
*Plasma IFN-ɣ*	0.19	**0.36**	0.12
*Plasma ACTH*	0.17	**0.35**	0.16
Hypothalamus IL-6	0.02	0.20	**0.49**
Hippocampus IL-4	0.02	−0.02	**0.42**
Hippocampus IL-10	0.16	−0.09	**0.37**
Hypothalamus IL-10	−0.26	0.04	**0.36**
*Hind paw IL-4*	0.00	0.03	**0.36**
Hippocampus IL-6	0.18	−0.22	**0.34**
Hippocampus IL-1β	0.03	0.00	0.29
Hippocampus IFN- ɣ	0.15	−0.33	0.33
Hippocampus MCP-1	−0.07	0.14	0.01
Hypothalamus IL-4	−0.21	0.05	−0.02
Hind paw CORT	0.29	0.29	0.02
Spleen IL-10	0.17	0.25	−0.05
Spleen IL-4	0.13	0.25	−0.06
Thymus CORT	0.18	−0.13	−0.07
Whole blood CORT	0.12	0.06	−0.18
*Plasma IL-4*	−0.02	−0.03	−0.05

Note. Values ≥ 0.34 are set in bold.

Previously reported measures^21^ are indicated in italics.

To investigate whether rats from the two colonies differentially relied on different networks (components) at different stages of disease, correlations between subjects’ component scores and their AA condition were performed (Fig. 5b–d). The *Endocrine/Immune Response in Peripheral Tissue* network (Component 1), was negatively correlated with the Adj/NA condition for both Charles River (r = −0.29, *p = *0.027) and Harlan (r = −0.36, *p* = 0.006) rats, and with the Adj/M-M condition for Charles River rats (r = −0.31, *p* = 0.018), but positively correlated with the Adj/M-M (r = 0.31, *p* = 0.018) and Adj/S condition (r = 0.79, *p* < 0.001) for Harlan rats (Fig. 5b). The *Proinflammatory Chemokine Response in Peripheral Tissue & Brain* network (Component 2) was positively correlated with the control condition for Harlan rats (r = 0.66, *p* < 0.001), but for Charles River rats, was positively correlated with the Adj/S condition (r = 0.55, *p* < 0.001) and negatively correlated with the Adj/NA condition (failed to develop AA, r = −0.59, *p* < 0.001) (Fig. 5c). Finally, for the *Central Balance of Pro-/Anti-Inflammatory Cytokines* network (Component 3), network for Charles River rats showed a negative correlation with the control condition (r = −0.36, *p* = 0.006) but positive correlations with the Adj/NA (r = 0.45, *p* < 0.001) and Adj/S (r = 0.72, *p* < 0.001) conditions, whereas for Harlan rats we found negative correlations with both the control (r = −0.44, *p* = 0.001) and Adj/M-M (r = −0.30, *p* = 0.025) conditions (Fig. 5d).

## Discussion

The present data provide insight into important differences in the endocrine and immune responses of SD rats from Charles River versus Harlan colonies to an inflammatory challenge. Building on our previous data showing that colony of origin impacts the AA disease course, HPA mediators, and cytokine profile in the plasma and hind paws^27^, the current analyses indicate that Harlan rats have alterations in the corticosterone profile in the hind paws and immune tissues, compared to Charles River rats. Moreover, Harlan rats have an unexpected pattern of cytokine activation in the brain, particularly the hypothalamus, generally showing higher cytokine levels under control conditions than with severe AA, while Charles River rats showed highest cytokine levels with arthritis. The CPCA analysis revealed that the differential responses in Charles River and Harlan rats are mediated by different endocrine-immune networks. With active AA, Harlan rats showed particular reliance on network 1, which included peripheral endocrine/immune and HPA activation (network 1), whereas Charles River rats relied primarily on activation of networks 2 and 3, showing enhanced involvement of chemokines and central cytokines. These findings highlight the power of probing for differential endocrine/immune networks activated under the same AA severity conditions, and demonstrate that, in contrast to the evaluation of single, independent parameters, a network approach leads to a deeper, more comprehensive understanding of possible differential underlying pathophysiology in subjects with clinically similar levels of inflammation. As well, our data have potential clinical implications, indicating that exploitation of colony differences may provide novel insights into the variable course and response to treatment that is characteristic of RA.

Females were used in this study due to the increased rates of autoimmune disorders in women compared to men^25^, and previously reported heightened sensitivity to experimentally induced arthritis in female rodents^26^. While estrous cycle stage/hormone levels could certainly impact immune measures^36^, findings of the current study, where 90% of rats were in diestrus, are consistent with previous data showing that chronic immune system activation results in estrous cycle disturbance and sustained diestrus to prevent ovulation^37, 38^.

We previously reported that AA increases plasma corticosterone levels in female rats from both Charles River and Harlan colonies^27^. Here we found that corticosterone changes in plasma do not necessarily parallel corticosterone changes in other tissues. While AA resulted in increased corticosterone across multiple tissues, differential patterns of response to CFA were detected by colony. For the spleen and popliteal lymph nodes, for example, while the ANOVA revealed an overall increase in corticosterone levels with AA, it appears that the higher corticosterone levels in the Adj/M-M condition were driven by changes in Harlan rats, while the higher corticosterone levels in the Adj/S condition were driven by changes in Charles River rats. This differential pattern suggests possible alterations in local regulation of corticosterone levels between colonies, potentially via inflammation-induced cleavage of CBG and increased corticosterone bioavailability^27^, local regeneration of corticosterone from dehydrocorticosterone (DHC) by 11β-hydroxysteroid dehydrogenase 1^39, 40^, or local corticosterone metabolism^41^. Each of these processes can be driven by pro-inflammatory cytokines, which generally increased to a greater extent in Charles River than Harlan rats. The relative lack of corticosterone increase in the hind paws of Harlan rats, the primary location of inflammation in the AA model, is also of interest, as corticosterone has significant anti-inflammatory properties [reviewed in^42^]. Decreased corticosterone availability at sites of inflammation such as the paws may be due in part to lower circulating CBG in Harlan, and may help explain the increased incidence of AA in Harlan compared to Charles River rats^27^.

A broad range of cytokines was measured in the hypothalamus, hippocampus, and spleen in order to gain a representative picture of the balance between pro-inflammatory (IL-1β, IFN-ɣ, TNF-α, IL-6) and anti-inflammatory (IL-4, IL-10, IL-6) cytokines (note: IL-6 has both pro- and anti-inflammatory properties). In addition, the chemokines MCP-1 and KC/GRO were measured as they are critically important for monocyte chemotactic activity and neutrophil recruitment^43^, respectively, and levels of TNF-α were measured, as they are thought to play a key role in neuroinflammation associated with RA^19^. Together, these analyses allowed for comprehensive profiling of the neuroinflammatory milieu in this model.

In the spleen, proinflammatory cytokines were found to increase, as expected, with CFA injection and/or arthritis onset. Statistically significant colony differences were detected in KC/GRO levels only, with lower cytokine levels in Harlan rats as a group, compared to Charles River rats. However, it appears that Harlan rats had a somewhat attenuated TNF-α response as well, particularly in those that developed severe AA, as the overall increase in TNF-α with increasing AA severity is clearly driven by the response in Charles River rats. Further, while all Charles River rats with mild-moderate and severe arthritis had detectable levels of IFN-ɣ, in Harlan rats 0% of mild-moderate AA cases and 40% of severe AA cases had detectable IFN-ɣ levels, and overall IFN-ɣ levels, while not analyzed statistically, appear to be attenuated in Harlan rats. While the joints are generally the inflammatory sites of most interest in arthritis models, the spleen, a critical secondary lymphoid organ, is also an important inflammatory site in this model. For example, arthritis can be passively transferred through inoculation of a healthy rat with spleen cells of an arthritic donor^44^. Rats with severe AA, as in the present study, develop enlarged spleens with grey/white spots, which has been identified as reactive granulomas that surround adjuvant material^44^. Thus an attenuated splenic cytokine response in Harlan rats is unexpected, particularly as these rats were shown to develop a more severe arthritic profile that in Charles River rats^27^.

In the hypothalamus, colony differences in the cytokine response to CFA injection indicated that, in general, Charles River rats responded as expected, with increased TNF-α, IFN-ɣ, and IL-6 production with the development of AA (Adj/M-M and/or Adj/S), while Harlan rats, remarkably, had high hypothalamic levels of these three cytokines under *control* conditions. Furthermore, 0% of Harlan rats had detectable hypothalamic levels of IL-1β or IL-10 with severe AA, as compared to 100% for IL-1β and 50% for IL-10 in Charles River rats. Importantly, chronic inflammation models, such as the AA model, induce inflammation in the central nervous system (CNS) via glial cell activation^45^. For example, intraplantar administration of CFA not only induced peripheral inflammation but also robust microglial cell activation and concomitant increased expression of proinflammatory cytokines (IL-1β, TNF-α, IL-6)^46^. Cytokines have a wide range of effects on homeostatic functions within the CNS and play a key role in sickness behaviors including anhedonia, anorexia, reduced quality of sleep, decreased motor activity, and social withdrawal^47^. In addition, cytokines likely modulate nociceptive responses including hyperalgesia and allodynia^48^. Finally, chronic neuroinflammation/increased cytokine levels in the brain is thought to occur in people with autoimmune or inflammatory disorders such as RA^19^. For example, treatment with anti-TNF-α agents including infliximab have been shown to effectively and quickly reduce fatigue in RA^23^. As well, neuroinflammation is thought to underlie the depression that may accompany this disorder^49^. Thus, the attenuated cytokine response in the brain of Harlan rats appears to reflect an inappropriate response to immune challenge, and might affect expression of sickness behaviors, which are critical for reorganizing the organism’s priorities and allowing it to cope with disease^50^. Furthermore, in light of the observed colony differences in the neuroimmune response to chronic inflammatory challenge, our results suggest that Charles River-derived rats would be the better subjects in models of neuroinflammation or inflammatory mediated depression.

To begin to explore possible mechanistic differences between colonies, heatmaps were constructed to integrate and provide an overview of cytokine activation patterns within the hypothalamus, hippocampus, and spleen. As a whole, in the hypothalamus, Charles River and Harlan rats had opposing cytokine pattern, with the highest cytokine levels in the Adj/M-M and Adj/S disease states for Charles River, and the highest cytokine levels in the control and Adj/NA states for Harlan rats. Similarly, in the hippocampus, Charles River rats overall had the highest cytokine levels with severe AA (Adj/S), while Harlan rats showed the highest overall cytokine levels in the control condition. By contrast, rats from the two colonies had more similar patterns of response in the spleen, with the highest cytokine levels generally occurring with severe AA (Adj/S). Integrating patterns of response in this heatmap data summary provides support for the suggestion that Charles River and Harlan rats activate different networks of cytokines in a unique and disease state-dependent manner.

Further investigation of cytokine networks through our novel CPCA analysis reinforced the suggestion of differential cytokine networks, providing evidence for differential underlying mechanism of AA in Charles River and Harlan rats. CPCA is the ideal statistical technique as it allows for networks of endocrine/immune parameters to be identified and related back to the independent variables being investigated. In addition, as the networks were based on the endocrine/immune variance constrained to that predictable by the independent variables, we were able to identify how these constrained variance-specific networks related to colony and AA status. While commonalities between the univariate results and the CPCA analysis are evident, similar to what was found with the heatmaps, CPCA facilitated a more global interpretation of this large dataset, revealing three components (i.e., endocrine/immune networks), that together represented 76.65% of the predictable variance (or 28.06% of the total variance) in the parameters examined.

Overall, with any level of AA (Adj/M-M, Adj/S), Harlan rats activated only network 1 (*Endocrine/Immune Response in Peripheral Tissue*), indicating increased reliance on peripheral endocrine/immune and HPA axis activation. Comparatively, with severe AA, Charles River rats activated networks 2 (*Proinflammatory Chemokine Response in Peripheral Tissue & Brain*) and 3 (*Central Balance of Pro/Anti-Inflammatory Cytokines*) indicating enhanced involvement of chemokines and central cytokines. These data suggest that under clinically similar levels of inflammation, Harlan and Charles River rats rely on/activate differential endocrine/immune pathways, suggesting differential underlying mechanisms and pathophysiology of disease. Of note, Harlan rats also show an unexpected pattern of activation of network 2 (*Proinflammatory Chemokine Response in Peripheral Tissue & Brain*) in the control, saline-injected condition. This heightened baseline activation of the proinflammatory chemokine-dominated network in both peripheral and central compartments may underlie, at least in part, the increased incidence and severity of AA observed in Harlan compared to Charles River rats^27^. Taken together, our findings highlight the potential utility of exploiting colony differences in the exploration of diseases with variably clinical presentation, such as RA, not only for understanding underlying disease mechanisms but also for the more targeted evaluation of therapeutic agents.

Moving forward, in the push to understand disease mechanisms and resiliency factors, and design precise intervention strategies, an understanding of networks of physiological and neurobiological variables is paramount. By exploring how seemingly similar disease states, such as clinically similar levels of inflammation in the current study, rely on different endocrine and immune networks, we can further the understanding of individual variability in disease. Gone is the idea of elucidating singular molecular targets, which would then form the basis of a universal treatment. Rather, personalized medicine, the optimization of treatment to the individual, taking into account genetics, environment, and lifestyle factors, is emerging as an important new approach in disease prevention, diagnosis, and treatment. While personalized medicine is becoming common in the cancer field, treatment efficacy for rheumatoid arthritis also varies widely and may benefit from increased investment in individualized care^51^.

## Materials and Methods

### Animals

Adult female Sprague Dawley rats (postnatal day [PND] 40 ± 2) were obtained from Charles River Laboratories International, Inc. (St. Constant, QC, Canada) and Harlan Laboratories, Inc. (Frederick, MD) (n = 29/vendor). Conditions in these colonies prior to arrival at the University of British Columbia (UBC) were previously reported^27^. At UBC, rats were pair-housed in a single colony room, and maintained under controlled temperatures (21–22 °C), on a 12:12 hour light/dark cycle. *Ad libitum* access to standard laboratory chow (Purina Laboratory Rodent Diet #5001, Delta, BC, Canada) and water was provided throughout the experiment. All procedures were in accordance with the National Institutes of Health Guide for the Care and Use of Laboratory Animals, and approved by the University of British Columbia Animal Care Committee.

### Adjuvant-Induced Arthritis (AA) Induction and Clinical Assessment

AA was induced as previously reported^27^. Briefly, on PND 55–60, rats received intradermal injections of either Complete Freund’s Adjuvant (CFA; n = 7–8/vendor/dose) or physiological saline (control; n = 6–7/vendor), at the base of the tail. CFA was prepared using *Mycobacterium tuberculosis* H37 RA (Difco laboratory, Detroit, MI) dissolved in incomplete Freund’s adjuvant^52^. Two initial doses of CFA were prepared – high, 1.2 mg/rat and low, 0.3 mg/rat were selected with the aim of identifying a dose that would result in arthritis onset in approximately 50% of rats. However, due to the previously observed heightened response of Harlan-derived rats to the low (0.3 mg) dose (40% of Harlan vs 0% of Charles River rats developed severe inflammation)^27^, two additional doses of CFA were added: 0.6 mg for Charles River and 0.2 mg for Harlan. Overall, the CFA doses selected resulted in low (Charles River: 0.3 mg; Harlan: 0.2 mg), moderate (Charles River: 0.6 mg; Harlan: 0.3 mg), and high (Charles River & Harlan: 1.2 mg) levels of AA, as previously reported^27^.

Following injection, rats were weighed and clinical scores, a baseline measure of AA severity, measured on days 6, 9, 11, 13, and 15 post-injection. Each of the four paws was scored on a 0–4 point scale, (0 = no inflammation, 1 = single focus of redness or swelling, 2 = two or more foci of redness or swelling, 3 = confluent but not global swelling, 4 = severe global swelling; total possible clinical score = 16). A clinical score ≥8 at any point during the study was classified as severe arthritis (Adj/S) whereas a clinical score ≥1 but < 8 was classified as mild-moderate arthritis (Adj/M-M). Rats were then categorized as: control (saline-injected), CFA-injected but no clinical signs of arthritis (Adj/NA), mild-moderate (Adj/M-M), or severe (Adj/S) AA. Levels of physiological parameters were then analyzed by colony and arthritis severity.

### Termination and tissue collection

At the peak of inflammation, day 16 post-injection, rats were quickly decapitated (<2 min; between 08:00 and 10:30 hr), and trunk blood collected. Draining popliteal lymph nodes, thymus, spleen, hypothalamus, hippocampus, front paws (at the level of the radiocarpal joint), and hind paws (at the level of the tibiotarsal joint) were collected and flash frozen in liquid nitrogen. All tissue samples were stored at −80 °C until assayed for protein and/or steroid levels. Vaginal lavage samples were collected and assessed cytologically for estrous cycle stage.

### Tissue homogenization

Tissue samples were homogenized in cold lysis buffer. Brain (hypothalamus, hippocampus) and spleen (0.15–0.20 g) were homogenized using the Omni Bead Ruptor 24 (Omni International, Kennesaw, GA). Hind paw were homogenized as reported previously^27^, with a separate aliquot removed for steroid extraction. Following homogenization, all tissue samples were centrifuged at 1,400 g for 10 min at 4 °C with supernatant collected for total protein quantification and cytokine analysis.

Prior to steroid measurements, organs (front paw, spleen, popliteal lymph nodes, thymus) were weighed to the nearest 0.1 mg, and plasma and whole blood measured to the nearest μl. Samples (with the exception of hind paws) were then homogenized in 3 volumes of water with a tissue homogenizer and diluted in 16 volumes of methanol. Front paws were weighed, pulverized in powdered dry ice with a mortar and pestle, and diluted in methanol. Hind paws were homogenized for cytokine analysis as above and an aliquot subsequently diluted in methanol. Similar to previous studies using brain tissue^53, 54^, we used protein levels in the hind paw to determine tissue weight, using a hind paw conversion factor calculated from samples from both Charles River and Harlan rats, at various severity states [*y* = 6.258*x*; where *y* = tissue mass (g), *x* = protein concentration (mg)]. After addition of methanol, all samples were thoroughly mixed and incubated overnight at 4 °C.

### Multiplex cytokine immunoassays and protein quantification

Cytokine levels were analyzed using a custom Meso Scale Discovery rat cytokine 8-plex panel, allowing for the measurement of IL-1β, IL-4, IL-6, IL-10, IFN-ɣ, KC/GRO (CXCL1), MCP-1 (CCL2), and TNF-α (catalog #: N05IA-1, MSD, Rockville, MD), plates read using a Sector Imager 2400, and data analyzed using the MSD Discovery Workbench software v. 4.0 (MSD, Rockville, MD). The lower limit of detection (LLOD) for the assays varied by plate and by analyte. The following LLOD ranges were observed (pg/mL): KC/GRO: 0.74–3.31; IFN-ɣ: 15.8–104; IL-10: 4.12–44.1; IL-1β: 6.03–23.5; IL-4: 1.94–8.26; IL-6: 35.4–144; MCP-1: 4.76–10.9; TNF-α: 1.73–12.6. Values falling below the LLOD were replaced with 0 pg/mL in all analyses and figures. Note: cytokines are presented in the following consistent order in Figs 2–4 : interleukins (IL-1β, IL-4, IL-6, IL-10), TNF-α, IFN-ɣ, and finally chemokines (KC/GRO, MCP-1), with undetectable cytokines omitted, when necessary.

Total protein levels were quantified in tissue homogenates using the Pierce Microplate BCA Protein Assay Kit (Pierce Biotechnology, Rockford, IL). Tissue cytokine levels were adjusted and reported as pg cytokine/mg of protein.

### Steroid extraction

Steroids were extracted from samples (plasma, thymus, spleen, popliteal lymph nodes, front paw, and hind paw) using solid phase extraction (SPE) with C_18_ columns, as previously described^55^. Homogenates were centrifuged at 3,000 g for 10 min at 2 °C, and supernatant aliquots (≤1.0 mL) were diluted with 10 mL deionized water before loading onto 500 mg C_18_ columns primed with 3 mL HPLC-grade ethanol and equilibrated with 10 mL deionized water. Columns were washed with 10 mL of 40% methanol^56^ and steroids were eluted with 5 mL of 90% methanol. Eluates were dried at 40 °C in a vacuum centrifuge (Thermo Scientific SPD111V).

Dried steroid residues were resuspended in steroid diluent with 5% ethanol to aid in resuspension^55^. Corticosterone recovery was determined by spiking tissue pools with known amounts of corticosterone, and comparing spiked and unspiked samples^54, 57^.

### Endocrine measures

Plasma ACTH levels and the steroid binding capacity of CBG were measured as previously reported^27^. Total (bound plus free) corticosterone levels were measured in plasma, thymus, spleen, popliteal lymph nodes, front paw, and hind paw using the ImmuChem Double Antibody Corticosterone ^125^I radioimmunoassay (RIA) kit (MP Biomedicals, LLC, Orangeburg, NY, USA), as previously described^58^. Cross-reactivity was 100% for corticosterone and less than 1% for all other tested steroids. The minimum detectable concentration of corticosterone was 1.56 pg/RIA tube, and the intra- and inter-assay coefficients of variation were <10.3% and 7.2%, respectively. All samples were measured in duplicate.

### Statistical analyses

Rats from the two colonies (Harlan, total n = 28; Charles River: total n = 29) were classified by injection condition (control, saline-injected: n = 6–7/colony; CFA-injected: n = 22–23/colony). Rats in the CFA condition were then further stratified by their AA severity in order to better compare levels of endocrine and immune markers in the two colonies under comparable arthritis conditions. Thus rats were categorized as: 1) failure to develop clinical signs of inflammation (Adj/NA [adjuvant-injected, no arthritis]; Charles River n = 14, Harlan n = 6); 2) mild-moderate AA (Adj/M-M; Charles River n = 3, Harlan n = 12), or severe AA (Adj/S; Charles River n = 4, Harlan n = 5). Note: the low n in the Adj/M-M and Adj/S conditions for Charles River rats was not by design but rather reflects the low incidence of mild-moderate and severe arthritis for Charles River rats, a small but clinically important group.

Data were first analyzed by analysis of variance (ANOVA) for the factors of colony and AA severity, followed by Fisher *post hoc* tests, as appropriate (IBM SPSS Statistics). Differences were considered significant at *p* ≤ 0.05. Significant ANOVA F statistic and *p* values are reported in the text; *post hoc p* values are reported in figure legends. Outliers (±2.5 SD > mean) were removed from the cytokine analyses, when appropriate. Corticosterone and cytokine data were not normally distributed and were transformed using the Blom rank-based normalization method^59^, prior to statistical analysis. Untransformed data are presented in the figures for clarity. Heatmaps were built on z-scored data, averaging cytokine levels by severity state and colony to demonstrate overall cytokine patterns. Heatmaps were generated using R statistical software.

CPCA (performed using Matlab) was utilized to identify networks of analytes collectively altered by AA across the various compartments analyzed. CPCA combines multivariate multiple regression and principal component analysis into a unified framework, and allows for the identification of networks (components) that are specifically predictable from the independent variables of interest^60, 61^. Briefly, CPCA involves first regressing the matrix of dependent variables (i.e., the z-score transformed endocrine and inflammatory measures) on the independent variables (i.e. colony and AA severity), resulting in a matrix of predicted scores reflecting the variance in endocrine and inflammatory measures that is predictable from colony and AA severity, referred to as the predictable variance. The second step in CPCA consists of a principal component analysis (PCA) on the predictable variance, which reveals multiple networks of endocrine and inflammatory measures that are directly predictable from the experimental manipulations. PCA is a data reduction technique that uses information about the dominant patterns of intercorrelation among a set of variables to reduce these variables into a smaller number of components (or networks) that best explain the variance in the dataset. A PCA on the predictable variance in the current study resulted in a number of components representing networks of endocrine and immune parameters that were stimulated (or not) in response to AA. The component loadings, listed in Table 1, indicate the degree to which each of the 44 variables examined (analytes in various tissue compartments) load onto/fit within each component. PCA solutions were separately rotated using Varimax with Kaiser normalization, and the number of components extracted was determined using scree plots^62^. In order to determine the degree to which the experimental conditions (vendor and inflammatory status) differed in terms of the networks activated in response to the immune challenge, correlations were computed between the experimental groups and the component scores from each of the extracted components (Fig. 5).
